# Persistent Headaches in an Avid Hiker: A Case of Chronic Coccidioidal Meningitis

**DOI:** 10.7759/cureus.42758

**Published:** 2023-07-31

**Authors:** Jared J Bies, Mariam Hassan, Swathi Prakash, Mateo Porres-Aguilar, Diego P Peralta

**Affiliations:** 1 Internal Medicine, Texas Tech University Health Sciences Center El Paso, El Paso, USA; 2 Infectious Diseases, Texas Tech University Health Sciences Center El Paso, El Paso, USA

**Keywords:** lentiform nucleus infarct, left temporal lobe infarct, fluconazole, leptomeningeal enhancement, coccidioidal meningitis, coccidiomycosis, disseminated coccidiomycosis, pulmonary coccidiomycosis, disseminated coccidioides, coccidioides immitis

## Abstract

The clinical presentation, diagnosis, treatment, and complications of coccidioidal meningitis caused by the dimorphic pathogenic fungus *Coccidioides* (*Coccidioides immitis *and* Coccidioides posadasii*) have been well documented in the literature. Despite the abundance of literature concerning this disease manifestation, it is not very commonly seen in clinical practice, delaying its diagnosis and treatment and leading to devastating neurological sequelae. Therefore, considering this disease process as a potential diagnosis in endemic areas is important for appropriate and timely treatment.

We present the case of a 26-year-old male who was found to have chronic coccidioidal meningitis on further investigation. The patient presented as a transfer for an abnormal head MRI with a three-month history of progressive occipital headaches and shortness of breath. Associated symptoms included transit vision loss, upper extremity numbness, night sweats, decreased appetite, and weight loss. Relevant risk factors were being a hiker and living in the southwest of Texas. The patient was started on empiric ceftriaxone and vancomycin. A repeat MRI showed leptomeningeal enhancement and acute infarcts in the left temporal lobe and lentiform nucleus. Cerebrospinal fluid (CSF) analysis showed pleocytosis with lymphocytic predominance, the presence of eosinophils, elevated protein level, and an extremely low glucose level. Further workup ruled out syphilis and tuberculosis.

Therefore, considering his clinical presentation, risk factors, and workup results, ceftriaxone and vancomycin were discontinued, and high-dose oral fluconazole was started, which produced a marked clinical response within the next 48 hours. A CT thorax showed findings suggestive of pulmonary coccidioidomycosis, and *Coccidioides* serology in both serum and CSF specimens returned positive.

## Introduction

A systemic fungal infection with *Coccidioides* spp. is typically acquired via inhalation of its arthroconidia found in soil [1]. It is endemic to the southwestern United States, northern Mexico, and Central and South America [2]. Coccidioidomycosis refers to the spectrum of diseases caused by the dimorphic fungus *Coccidioides*. It has been reported to involve almost all organ systems; however, pulmonary disease is the most common clinical manifestation [1]. It is often a benign self-limited illness; nonetheless, it can disseminate outside of the lungs.

Coccidioidal meningitis (CM) is a relatively uncommon and severe form of coccidioidomycosis with complications if left untreated [2]. It is a condition that affects between 200 to 300 persons annually within the endemic areas of the United States [3]. Affected individuals may solely complain of a headache without any other symptoms, which challenges a clinician with a low index of suspicion. CM is more likely to occur in immunocompromised individuals, for example, those with HIV infection, but it can also manifest in immunocompetent individuals [4,5].

This article was previously presented as a meeting abstract poster presentation at the 2023 South Regional Meeting on February 2, 2023, at the InterContinental New Orleans in New Orleans, Louisiana, USA. 

## Case presentation

**Figure 1:**
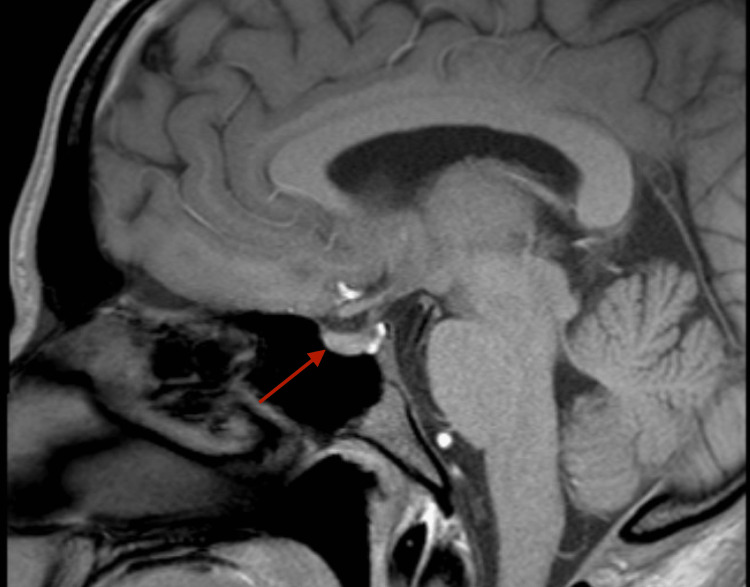
MRI Sella (pituitary with + without contrast)

A 26-year-old male with glucose-6-phosphate dehydrogenase (G6PD) deficiency, attention deficit hyperactivity disorder (ADHD), and depression presented as a hospital transfer after an abnormal outpatient head MRI showed findings concerning for a pituitary adenoma and an area of restriction on diffusion-weighted imaging on the left temporal area (Figure 1).

Three months prior to this, the patient presented to a local emergency department for chest pain and shortness of breath. After a negative work-up in the emergency department, the patient was discharged home. Following discharge, the patient reported having continued shortness of breath with the development of night sweats and an occipital headache that radiated to bilateral temporal areas of his head. The patient stated that his headache was exacerbated by neck flexion. The patient’s primary care provider (PCP) attributed these symptoms to the patient’s home medications, dextroamphetamine/amphetamine and duloxetine, so they were discontinued. Despite discontinuing dextroamphetamine/amphetamine and duloxetine, the patient’s symptoms persisted and progressed with the development of intermittent blurry vision with occipital/bitemporal headaches.

The patient stated that he also had two episodes of numbness on his right upper and lower extremities that only lasted a few seconds and self-resolved. In association with these symptoms, the patient also claimed to have lost 20 pounds in a month, secondary to having a low appetite. Due to his progressive symptoms, his PCP ordered labs to work up possible causes of his symptoms. Lab work returned with an elevated prolactin and testosterone level. This prompted his PCP to order an MRI of the head regarding high suspicion for a potential prolactinoma. The findings were concerning for potential pituitary adenoma and abnormalities of the left temporal lobe. Concerning significant social history, the patient stated that he worked in a military base located in Southwest Texas and was an avid hiker. On initial presentation, the patient was vitally stable. The only positive finding on examination was pain on neck flexion. The patient was started on empiric ceftriaxone and vancomycin. The initial laboratory workup revealed the following (Table 1).

**Table 1:** Initial laboratory workup. AST: Aspartate Transaminase, ALT: Alanine Transaminase, PT: Prothrombin Time, PT-INR: Prothrombin Time-International Normalized Ratio, APTT: Activated Partial Thromboplastin Time, SED Rate: Erythrocyte Sedimentation Rate, CRP: C-Reactive Protein, LDH: Lactate Dehydrogenase.

	Normal Range	Results
Serum Tests		
White blood cells	4.5-11.0 x 10^3^/μL	11.01 x 10^3^/μL
Red blood cells	3.5-5.5 x 10^6^/μL	4.50 x 10^6^/μL
Hemoglobin	12.0-15.0 g/dL	14.2 g/dL
Hematocrit	38.0-47.0%	42.5%
Platelets	150-450 x 10^3^/μL	282 x 10^3^/μL
Sodium	135-145 mmol/L	135 mmol/L
Potassium	3.5-5.1 mmol/L	4.2 mmol/L
Chloride	98-107 mmol/L	103 mmol/L
Bicarbonate	22-30 mmol/L	26 mmol/L
Glucose	74-106 mg/dL	110 mg/dL
Blood urea nitrogen	7-20 mg/dL	17 mg/dL
Creatinine	0.52-1.04 mg/dL	1.10 mg/dL
AST	17-59 IU/L	31 IU/L
Alkaline Phosphate	38-126 IU/L	71 IU/L
ALT	0-50 IU/L	33 IU/L
PT	11.8-14.8 sec	13.6 sec
PT-INR	0.9-1.1 sec	1.0 sec
APTT	23.3-38.6 sec	30.5 sec
SED Rate	0-14 mm/hr	57 mm/hr
CRP	0-1 mg/dL	0.65 mg/dL
LDH	120-246 IU/L	151 IU/L

**Figure 2:**
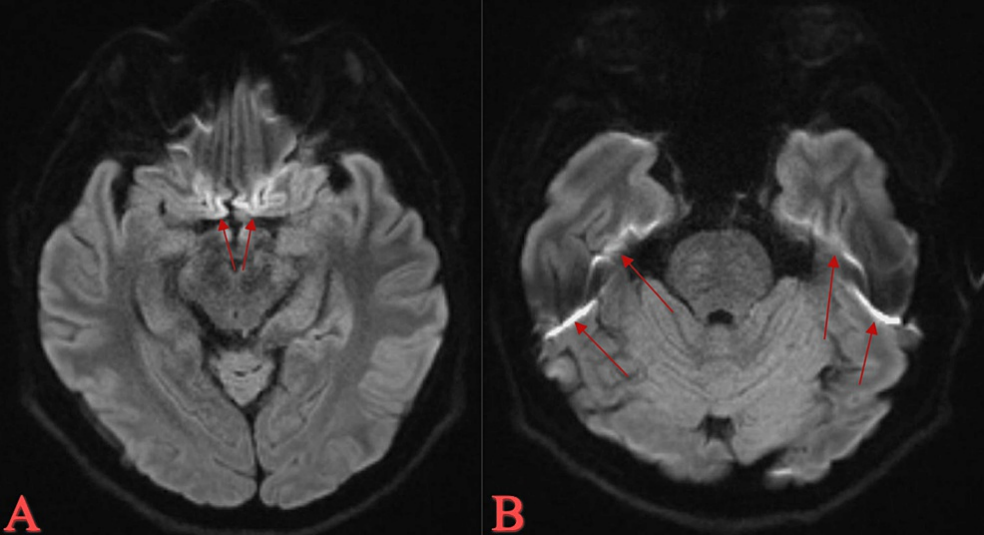
MRI T1 Leptomeningeal enhancement with basilar predominance (A) and of temporal lobes (B).

**Figure 3:**
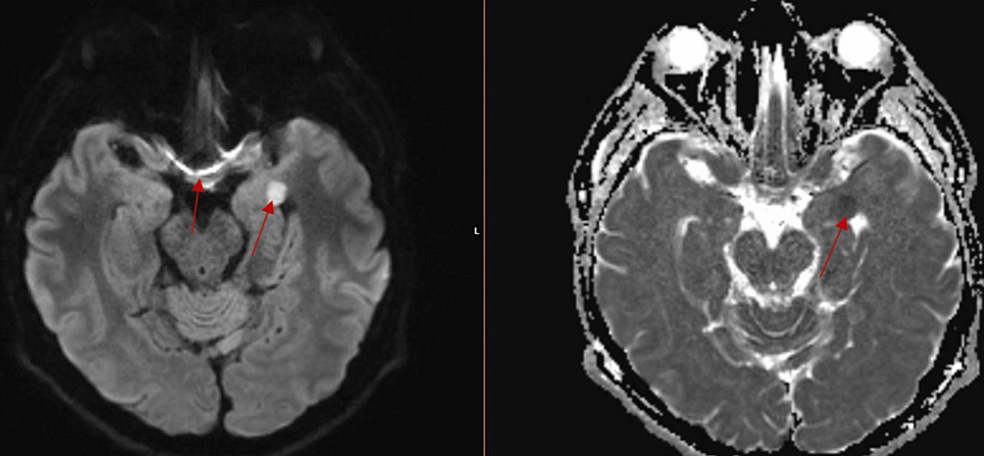
MRI T1/T2 Leptomeningeal enhancement with basilar predominance. Acute infarct in the left temporal lobe.

**Figure 4:**
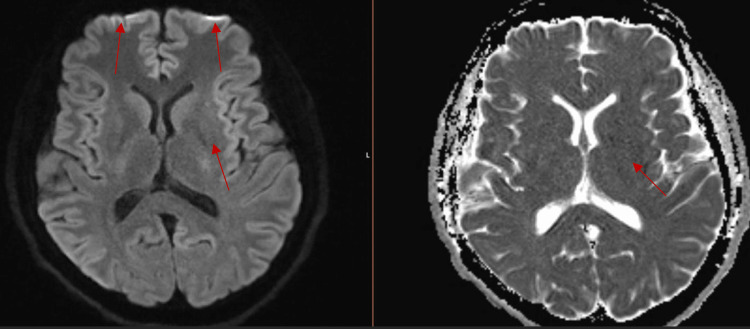
MRI T1/T2 Leptomeningeal enhancement of frontal lobes. Acute infarct in the left lentiform nucleus.

**Figure 5:**
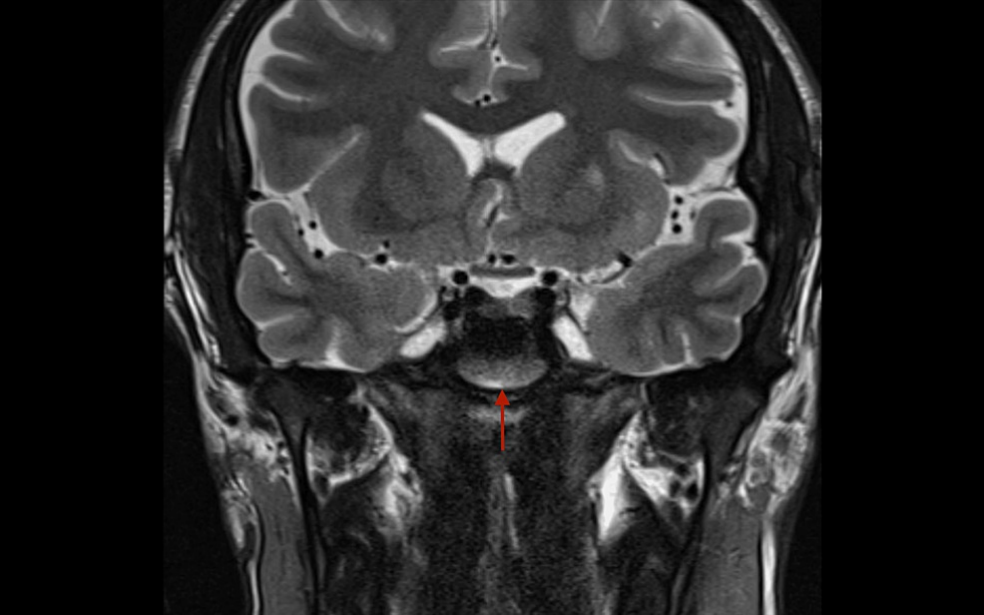
MRI head showing faint intertriginous hypo-enhancing area in the pituitary.

On admission, MRI head showed leptomeningeal enhancement of temporal lobes with basilar predominance, acute infarcts in the left temporal lobe and lentiform nucleus, and faint intertriginous hypo-enhancing area in the pituitary (Figures 2-5).

Cerebrospinal fluid (CSF) analysis showed pleocytosis with lymphocytic predominance and the presence of atypical lymphocytes and eosinophils. Protein level was elevated, and glucose level was extremely low (Table 2).

**Table 2:** Cerebrospinal fluid analysis.

	Normal Range	Results
Cerebrospinal Fluid		
Color	Colorless	Colorless
Appearance	Clear	Clear
Red blood cells	<5 /µL	10 /µL
White blood cells	<5 /µL	440 /µL
Neutrophils	0%	5%
Lymphocytes	0%	58%
Eosinophils	0%	2%
Macrophages	3-37%	14%
Atypical Lymphocytes	0%	21%
Glucose	40-70 mg/dL	<20 mg/dL
Protein	12-60 mg/dL	202 mg/dL

These findings were concerning for coccidioidomycosis, tuberculosis, and syphilis. Serum rapid plasma reagin (RPR), cerebrospinal fluid (CSF) venereal disease research laboratory (VDRL) test, CSF acid-fast stain and culture, CSF meningitis panel, CSF mycobacterium tuberculosis (MTB) complex polymerase chain reaction (PCR), and serum QuantiFERON-TB serum test were all negative, ruling out syphilis and tuberculosis. Due to high suspicion for coccidioidomycosis given the patient’s clinical presentation and work-up results, ceftriaxone and vancomycin were discontinued, and the patient was started on empiric high dose fluconazole 1 gram orally daily.

**Figure 6:**
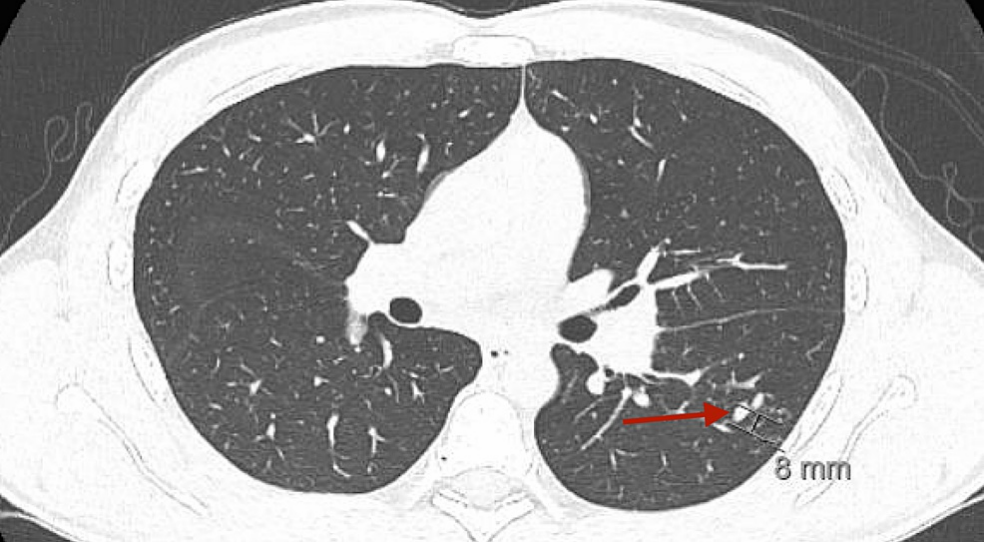
CT Thorax without contrast: elongated nodular lesion in the apical segment of the left lower lobe.

**Figure 7:**
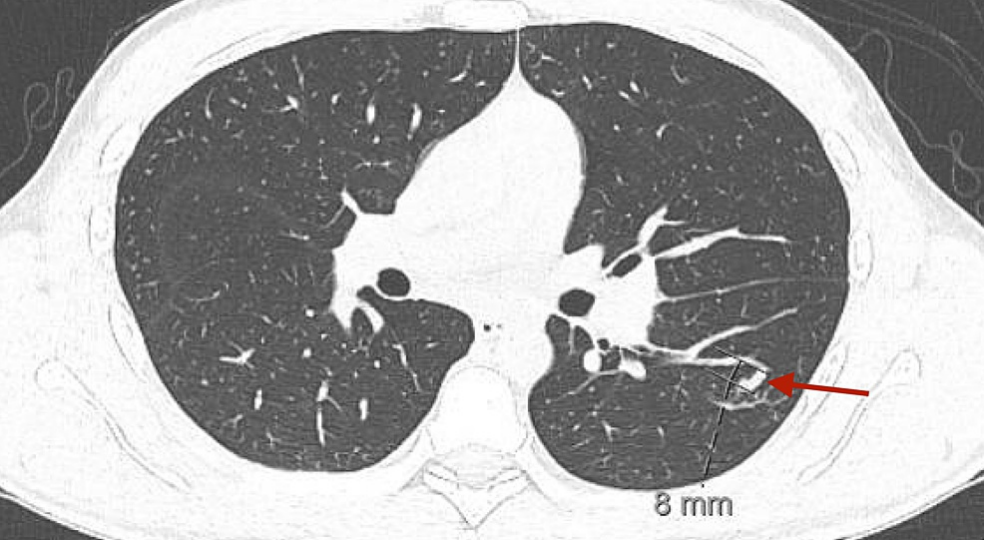
CT Thorax without contrast: elongated nodular lesion in the apical segment of the left lower lobe.

**Figure 8:**
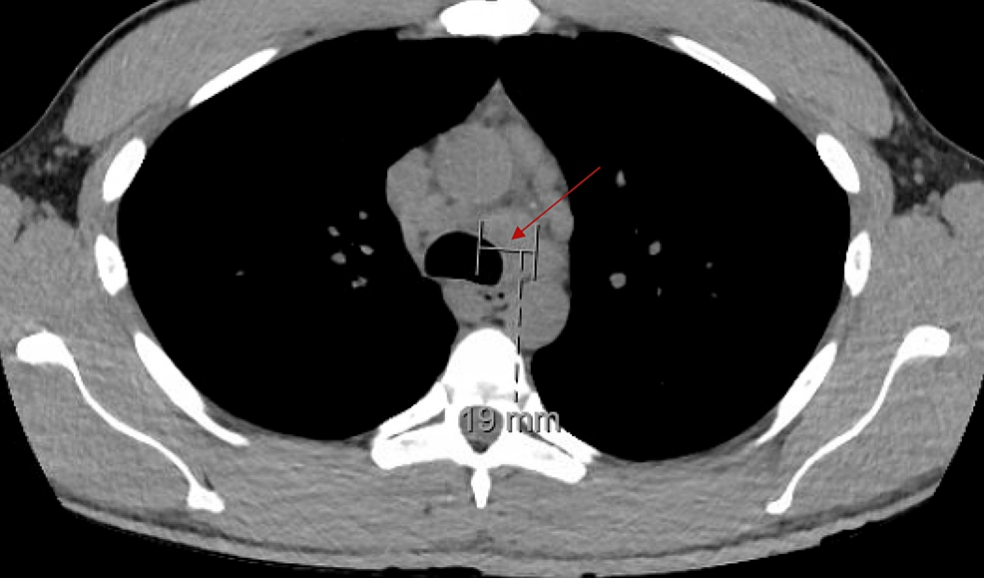
CT Thorax without contrast: mediastinal adenopathy with largest lymph node measuring 19 mm.

A CT thorax without contrast was significant for two nodular lesions in the apical segment of the left lower lobe with adjacent tree-in-bud opacities and mediastinal adenopathy with the largest lymph node measuring 19 mm, suggestive of pulmonary coccidioidomycosis (Figures 6-8).

These findings have been well-documented to be suggestive of pulmonary coccidioidomycosis [1,6]. *Coccidioides* serology in both serum and CSF returned positive (Table 3).

**Table 3:** Serum and cerebrospinal fluid (CSF) Coccidioides serology by immunodiffusion and complement fixation.

	Complement Fixation Titer	Immunodiffusion
Serum	1:64	Positive
CSF	1:4	Positive

The patient’s headache gradually resolved while being on the fluconazole trial within 48 hours. The patient was recommended to continue fluconazole 1 gram orally daily for 4-6 months, then decrease to 400 mg daily for lifelong treatment. The patient was also advised for periodic monitoring of *Coccidioides* serology and imaging studies. It was also recommended to obtain an immunodeficiency workup as a possible contributing risk factor for the underlying infection. Immunodeficiency workup completed prior to discharge showed elevation in immunoglobulin E, while all other workup results were normal (Table 4).

**Table 4:** Immunodeficiency workup.

	Normal Range	Results
Serum Tests		
C3	88-165 mg/dL	137 mg/dL
C4	14-44 mg/dL	27 mg/dL
Immunoglobulin A	47-310 mg/dL	131 mg/dL
Immunoglobulin E	<=114 kU/L	1391 kU/L
Immunoglobulin G	600-1640 mg/dL	1328 mg/dL
Immunoglobulin M	50-300 mg/dL	143 mg/dL
HIV ½ 4^th^ Generation	Negative	Negative

## Discussion

CM is an extrapulmonary manifestation of coccidioidomycosis that occurs when *Coccidioides* spherules migrate to the central nervous system (CNS). A persistent headache is the most common complaint [2]. Affected individuals can also have cranial nerve deficits, gait disturbances, or changes in mental status [7]. MRI results vary substantially, but basilar leptomeningeal enhancement is a classic finding [8]. Lymphocytic pleocytosis, eosinophilia, hypoglycorrhachia, and elevated protein level are seen in CSF analysis [7]. The diagnosis can be made by microscopic identification of *Coccidioides *spherules, fungal isolation in culture, or serologic testing [9]. Interestingly, our patient’s presentation, workup results, and strong suspicion of CM allowed a prompt initiation of antifungal therapy and prevented catastrophic CNS sequelae that are generally implicated throughout virtually accessible literature.

Prompt diagnosis of CM is critical, considering that meningitis represents the most severe and frequent manifestation of disseminated disease following initial pulmonary infection [3]. The complications of undiagnosed CM can lead to deleterious consequences, such as aneurysm formation in the CNS [10]. The progression of this complication can result in aneurysm rupture and consequent subarachnoid hemorrhage with clinical manifestations of altered mental status and hydrocephalus [11]. The pathogenesis of fungal aneurysm rupture is via migration of mast cells at the wall of the arterial vessel in response to the fungus causing a vast inflammatory response. CM can also lead to intracranial vasospasm due to vasculitic or subacute fibrotic changes requiring percutaneous transluminal angioplasty [12]. In rare cases, there can be co-infection with other pathogens, such as *Treponema pallidum*, which can cause severe brain damage and neurologic disability [13]. CM can present with relatively benign symptoms that can be misdiagnosed as chronic migraines or cognitive impairment and lead to mistreatment for years.

Before introducing azole therapy, CM was treated with intrathecal (IT) amphotericin B. However, it was associated with many severe side effects. High-dose fluconazole is now considered first-line therapy [2]. Fluconazole therapy appears to suppress rather than cure coccidioidomycosis meningeal disease, and evidence demonstrates a high relapse rate when azoles are reduced in dose or discontinued in CM patients [2]. Therefore, it is recommended to continue azole therapy for life in patients with CM who achieve remission and do not deteriorate on treatment [2]. IT amphotericin B, itraconazole, voriconazole, posaconazole, or isavuconazole are considered in patients who develop a refractory infection after fluconazole therapy. In a single-center retrospective chart review study, a high failure rate of initial fluconazole therapy for CM was found, supporting the need for further research of alternative treatments [14]. A case series of three patients with refractory CM showed clinical improvement with isavuconazole, used as salvage therapy [15]. Prompt serologic CM diagnosis through Coccidioides antigen testing, serum and CSF antibodies by immunodiffusion and complement fixation, and a promising new coccidioidal chitinase-1 (CTSI) inhibition enzyme-linked immunosorbent assay (ELISA) can aid in timely treatment to prevent clinical deterioration [16].

Predisposing risk factors for disseminated coccidioidomycosis in the immunocompromised include HIV infection and the use of corticosteroids and immunotherapy, as reported in the retrospective study of 71 cases of CM and brain abscesses by Drake and Adam [17]. HIV-infected individuals are susceptible to developing CM in non-endemic areas during antiretroviral therapy as a manifestation of immune reconstitution inflammatory syndrome [18]. On the contrary, immunocompetent individuals having disseminated coccidioidomycosis in the form of CM are rare. Few reports demonstrate rapidly disseminated infections in immunocompetent patients. Fatima et al. reported an immunocompetent individual with early manifestations of CM who received early treatment that prevented neurological sequelae [19].

Having high suspicion for coccidioidomycosis in endemic areas is important. However, in non-endemic areas, clinical suspicion should also be present. Most of these cases occur in individuals or groups who visit or temporarily relocate to endemic areas and seek medical care after returning to their permanent residence [20]. Thus, it is important to educate travelers and healthcare providers in non-endemic areas about coccidioidomycosis so that the diagnosis and treatment will not be delayed [20].

Imaging findings in acute/chronic CM show areas of focal enhancement in the cervical subarachnoid space, basilar, Sylvian, and interhemispheric cisterns representing focal collections of the organism [8]. Deep infarcts and communicating hydrocephalus are also associated findings. The neuroimaging of CM also shows hydrocephalus, periventricular edema, meningeal enhancement abnormalities, leptomeningitis, pachymeningitis, cranial nerve enhancement, and vasculitic abnormalities [21]. Intense enhancement regions tend to decrease during therapy. Decompensated patients with severe presentations of CM often present with hydrocephalus and require placement of a ventriculoperitoneal shunt [22]. A case series of five children showed hydrocephalus as a common complication of severe disseminated CM [23]. This complication can be associated with or a result of cerebral vasculitis caused by the inflammatory component of the CM infection.

Our patient presented with a known G6PD deficiency in correspondence to additional potential undiagnosed immunodeficiencies, which ultimately could have served as a significant risk factor for disseminated coccidioidomycosis in the form of CM. G6PD deficiency can lead to reduced nicotinamide adenine dinucleotide phosphate oxidase activity in phagocytes, resulting in immunodeficiency, and can lead to bacterial, viral, and fungal infections [24,25].

This case presents an indolent course of CM in a young immunocompetent individual with timely treatment that prevented severe neurological deterioration. The patient had benign symptoms for 3 months which prompted further workup. MRI head showed leptomeningeal enhancement with basilar predominance and acute infarcts in the left temporal lobe and lentiform nucleus, consistent with documented findings of CM. CSF analysis was consistent with findings of a fungal infection. High-dose fluconazole was started per Infectious Diseases Society of America (IDSA) guidelines with clinical improvement [2]. Serum and CSF antibodies by immunodiffusion and complement fixation were positive for *Coccidioides,* supporting the diagnosis.

## Conclusions

It is imperative to place CM on the differential list for even a seemingly innocuous complaint, such as a headache, in regions where *Coccidioides* is native. A retrospective study of the patient’s findings helps identify our own limitations in providing appropriate care. Clinicians are encouraged to acquaint themselves with the epidemiology, symptomatology, and relevant laboratory values that commonly accompany this condition with the potential for detrimental consequences. 
